# Azilsartan ameliorates apoptosis of dopaminergic neurons and rescues characteristic parkinsonian behaviors in a rat model of Parkinson’s disease

**DOI:** 10.18632/oncotarget.15732

**Published:** 2017-02-25

**Authors:** Qing Gao, Zhou Ou, Teng Jiang, You-Yong Tian, Jun-Shan Zhou, Liang Wu, Jian-Quan Shi, Ying-Dong Zhang

**Affiliations:** ^1^ Department of Neurology, Nanjing First Hospital, Nanjing Medical University, Nanjing, PR China

**Keywords:** renin-angiotensin system, angiotensin II, Parkinson’s disease, apoptosis, azilsartan, Gerotarget

## Abstract

Loss of dopaminergic neurons within the substantia nigra (SN) is a pathological hallmark of Parkinsons disease (PD), which leads to the onset of motor symptoms. Previously, our *in vitro* studies revealed that Angiotensin II (Ang II) induced apoptosis of dopaminergic neurons through its type 1 receptor (AT1R), but these findings needed to be confirmed via animal experiments. Here, using a rotenone-induced rat model of PD, we observed an overactivation of Ang II/AT1R axis in the SN, since Ang II level and AT1R expression were markedly increased. Furthermore, we provided *in vivo* evidence that Ang II directly elicited apoptosis of dopaminergic neurons via activation of AT1R in the SN of rats. More importantly, we showed for the first time that oral administration of azilsartan, a newly developed AT1R blocker approved by the U.S. Food and Drug Administration for hypertension treatment, rescued the apoptosis of dopaminergic neurons and relieved the characteristic parkinsonian symptoms in PD rats. These results support the application of AT1R blockers in PD therapy, and strengthen the notion that many therapeutic agents may possess pleiotropic action in addition to their main applications.

## INTRODUCTION

The renin-angiotensin system (RAS) is widely accepted as an important modulator within circulation system, regulating the blood pressure and maintaining the sodium and water homeostasis [1]. Angiotensin II (Ang II) is the major effector of RAS, and most of its classical peripheral functions were medicated by Ang II type 1 receptor (AT1R) [2]. Recently, numerous studies have demonstrated that independent RAS existed in many other tissues and organs including the brain [3–5]. The locally formed Ang II plays an important functional role in the central nervous system [6] and has also been linked to several neurological disorders such as Parkinson's disease (PD) [7].

PD is well recognized as the second most common neurodegenerative disorder after Alzheimer's disease, and is mainly attributed to a progressive loss of dopaminergic neurons in the substantia nigra (SN) [8]. Decreased viability of dopaminergic neurons leads to the onset of rigidity, bradykinesia, resting tremor and postural instability [8]. A number of studies have suggested a potential relationship between hyperactivated RAS within the basal ganglia and PD progression, since the level of Ang II was significantly increased in the SN and striatum of PD patients. This finding was also supported by a series of *in vitro* experiments showing that Ang II, *via* AT1R, induced loss of dopaminergic neurons and thus contributed to the pathogenesis of PD [6]. Furthermore, our previous studies using CATH.a cell revealed that Ang II-triggered loss of dopaminergic neurons was attributed to cell apoptosis [9, 10]. However, all these *in vitro* findings need to be further confirmed *via* animal experiments.

In this study, we confirm that Ang II elicits apoptosis of dopaminergic neurons through an AT1R-dependent manner in the SN of normal rats. More importantly, we provide the first evidence that oral administration of azilsartan, a newly developed AT1R blocker approved by the U.S. Food and Drug Administration (FDA) for hypertension treatment [11], rescues the apoptosis of dopaminergic neurons and relieves the characteristic parkinsonian symptoms in a PD model induced by rotenone. These results support the application of AT1R blockers in the therapy of PD, and strengthen the notion that many therapeutic agents may possess pleiotropic action in addition to their main applications.

## RESULTS

### Ang II/AT1R axis is upregulated in the SN of a rat model of PD

**Figure 1:**
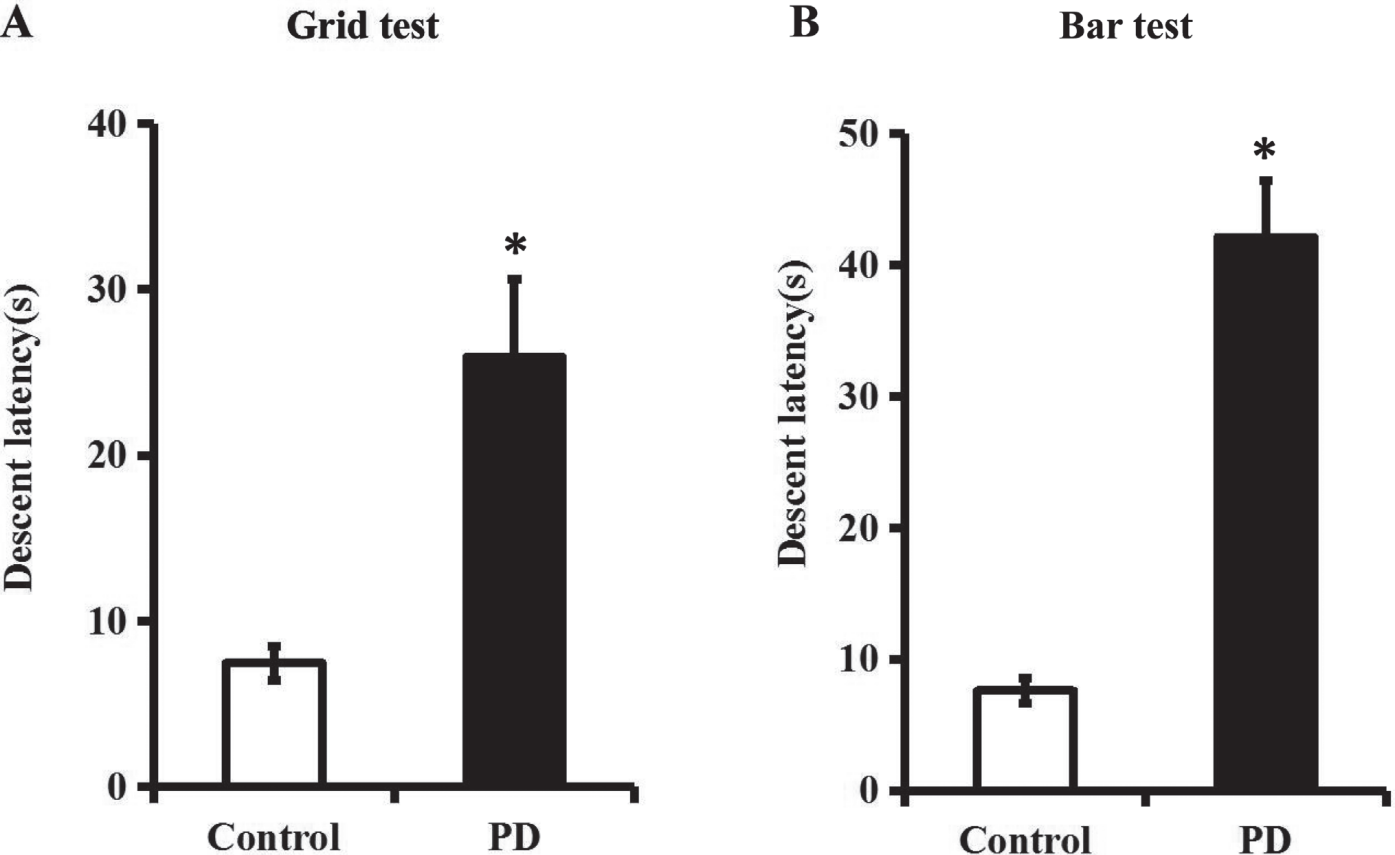
Descent latency is significantly prolonged in a rat model of PD A rat model of PD was established using a four-week infusion of rotenone (2.5 mg/kg/d). **A**. and **B**. Descent latency of control and PD group in the grid test and the bar test was recorded. *n* = 6 per group. Data were analyzed by independent samples t test. Columns represent mean±SD. *P < 0.05 *vs*. control group.

**Figure 2:**
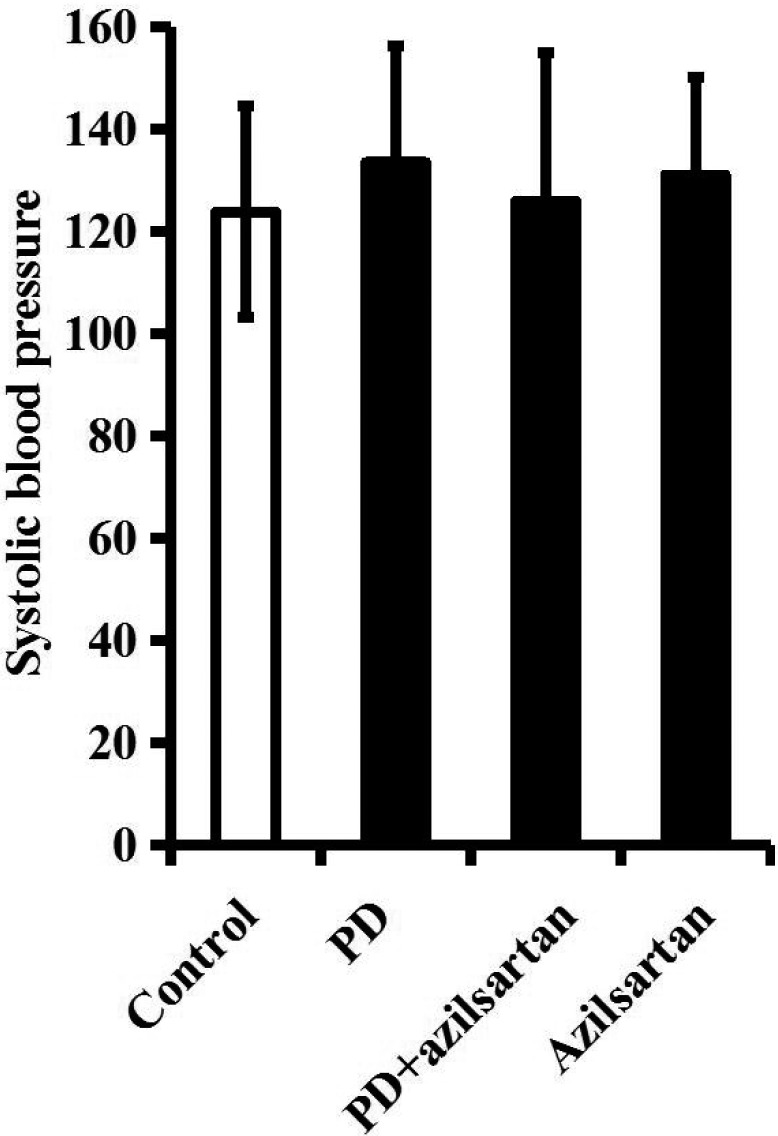
Rotenone and (or) azilsartan treatment does not influence the SBP of rats The SBP of rats in control group, PD group, PD + azilsartan (5 mg/kg/d) group or control + azilsartan (5 mg/kg/d) group was tracked by a tail cuff method. *n* = 6 per group. Data were analyzed by one-way ANOVA followed by Tukey's post hoc test. Columns represent mean±SD.

In this study, a rat model of PD was successfully established using a four-week infusion of rotenone (2.5 mg/kg/d) [12], since chronic rotenone treatment significantly prolonged descent latency when compared with that in the control group (Figure 1, *P* < 0.05). Meanwhile, the systolic blood pressure (SBP) of rats was not altered during this process (Figure 2, *P* > 0.05).

**Figure 3:**
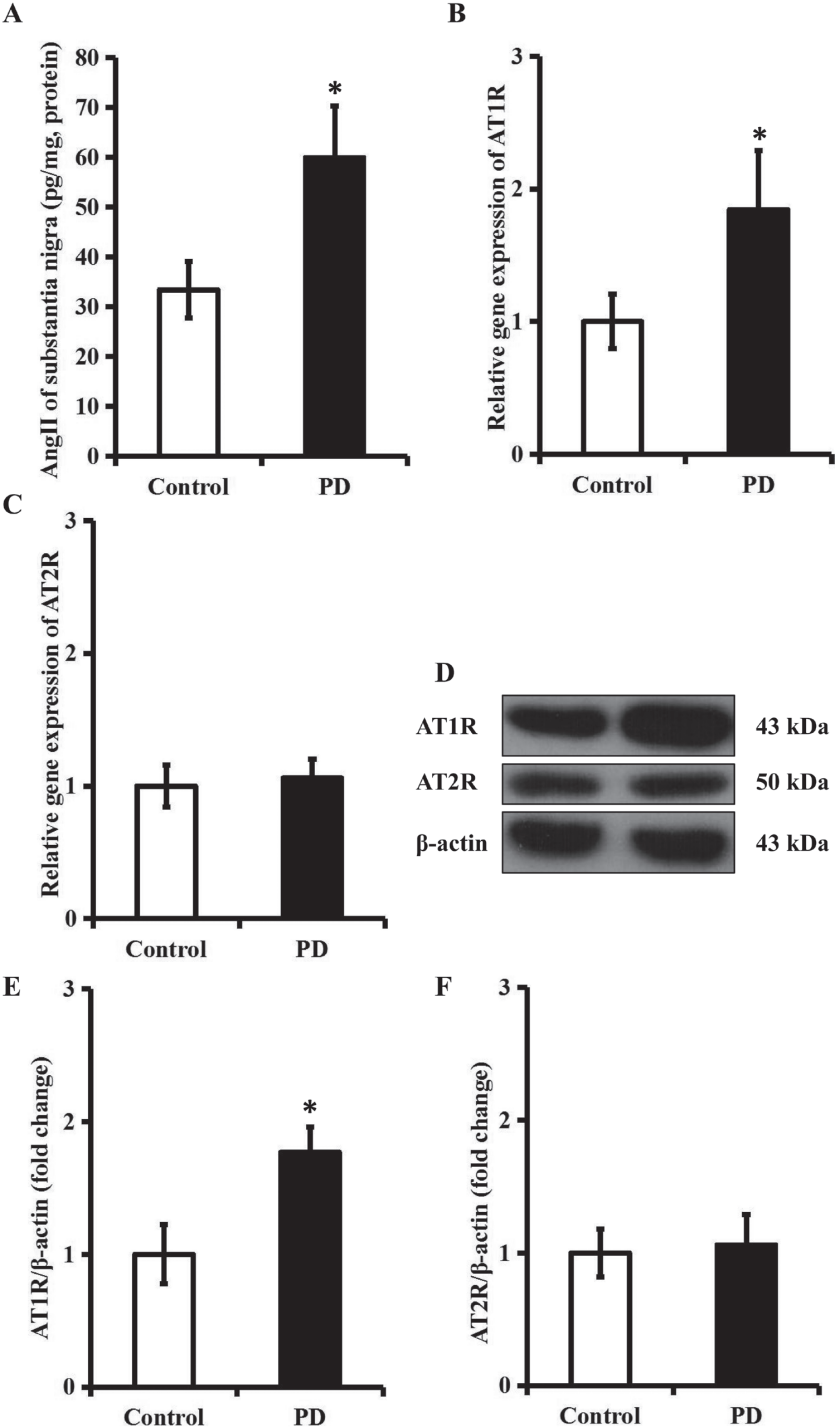
Ang II/AT1R axis is upregulated in the SN of a rat model of PD The rat model of PD was established using a four-week infusion of rotenone (2.5 mg/kg/d). **A**. The level of Ang II in the SN in the control and PD group. **B**. and **C**. The mRNA level of AT1R and AT2R in the SN in control and PD group. **D**.-**F**. The protein level of AT1R and AT2R in the SN in control and PD group. *n* = 6 per group. Data were analyzed by independent samples t test. Columns represent mean±SD. *P < 0.05 *vs*. control group.

To determine whether Ang II and its receptors were involved in the pathogenesis of PD, we first used a commercial enzymatic immunoassay (EIA) kit to detect the level of Ang II in the SN of PD rats. As revealed by Figure 3A, the level of Ang II in the SN of PD rats was approximately increased by 80% when compared with that of control rats (*P* < 0.05). Afterwards, we employed quantitative real-time polymerase chain reaction (qRT-PCR) and western blotting to investigate whether the expression of Ang II receptors was also changed in the SN of this model. As indicated by Figure 3B-3F, AT1R mRNA and protein levels in the SN of PD rat models were respectively elevated by 84.4% and 76.9% in comparison to control group (*P* < 0.05), whilst no obvious changes of Ang II type 2 receptor (AT2R) level were observed (*P* > 0.05). These findings implied that Ang II/AT1R axis might be involved in the pathogenesis of PD.

### Exogenous Ang II triggers apoptosis of dopaminergic neurons *via* an AT1R-dependent manner in the right SN of rats

**Figure 4:**
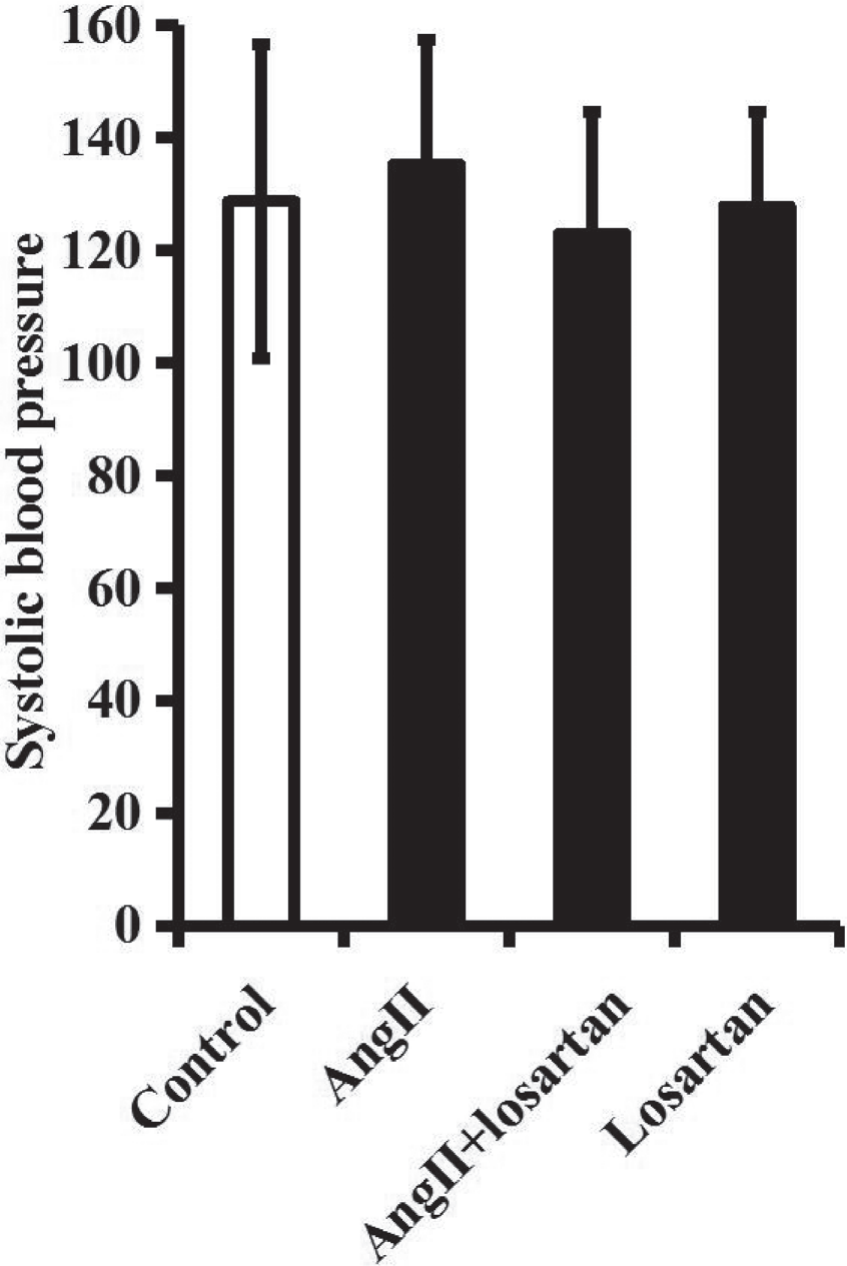
Ang II and (or) losartan treatment does not influence the SBP of rats The SBP of rats in control group, Ang II (60 ng/h) group, Ang II (60 ng/h) + losartan (200 ng/h) group or losartan (200 ng/h) group was tracked by a tail cuff method. *n* = 6 per group. Data were analyzed by one-way ANOVA followed by Tukey's post hoc test. Columns represent mean±SD.

To further determine the relationship between Ang II/AT1R axis and PD pathogenesis, exogenous Ang II was directly infused into the right SN of normal rats for one week. During this process, the SBP of rats was not significantly altered (Figure 4).

**Figure 5:**
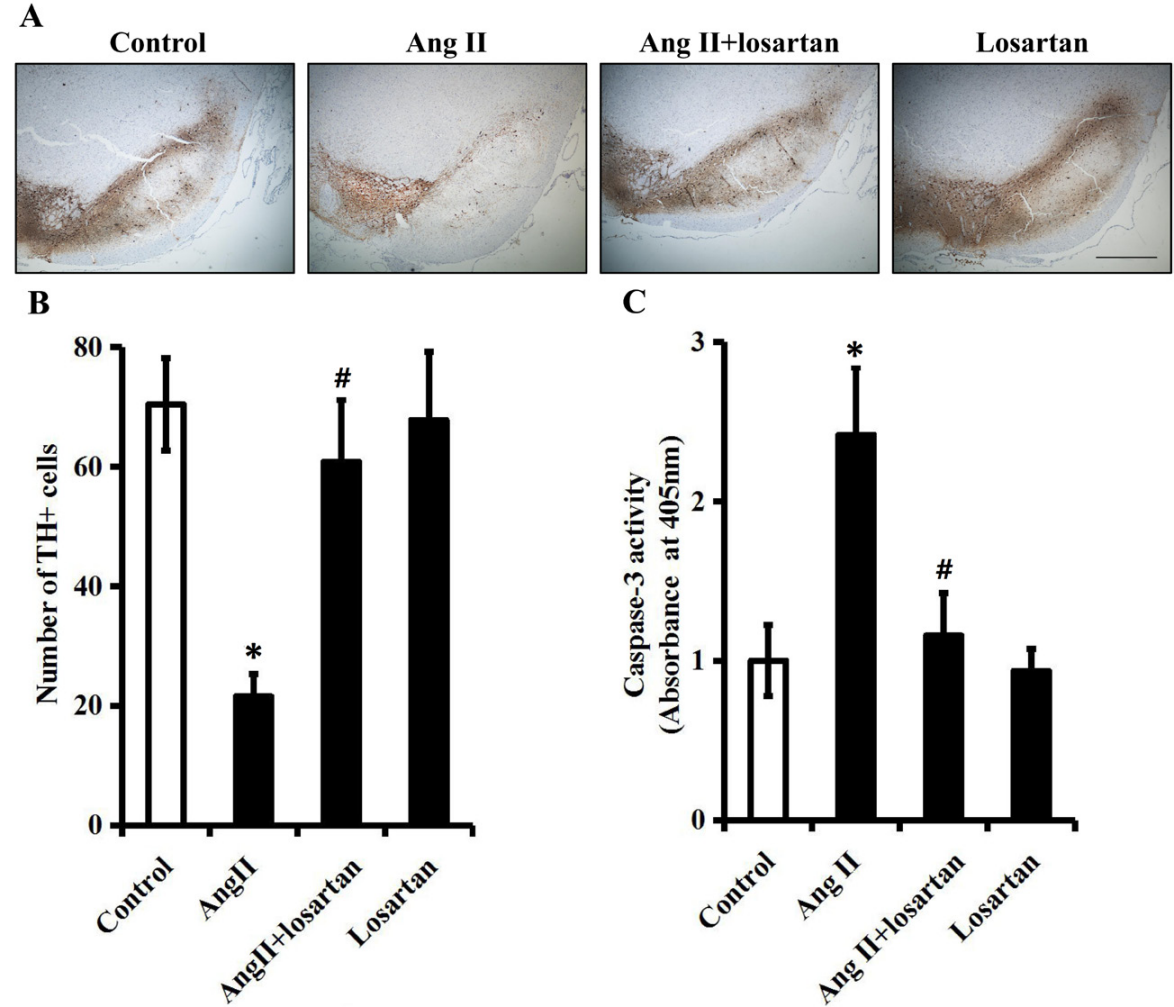
Exogenous Ang II triggers apoptosis of dopaminergic neurons *via* an AT1R-dependent manner in the right SN of rats The right SN of rats were infused with vehicle, Ang II (60 ng/h), Ang II (60 ng/h) + losartan (200 ng/h) or losartan (200 ng/h) for one-week. **A**. TH-immunoreactive neurons in the right SN of each group. Scale bar: 500 μm. **B**. Quantitative analysis of TH-positive neurons in the right SN of each group. **C**. The activity of caspase-3 in the right SN of each group was measured by a colorimetric assay kit. *n* = 6 per group. Data were analyzed by one-way ANOVA followed by Tukey's post hoc test. Columns represent mean±SD. *P < 0.05 *vs*. control group, #*P* < 0.05 *vs*. Ang II group.

First, immunohistochemical staining was used to examine the effect of exogenous Ang II on dopaminergic neurons in the right SN of rats. As indicated in Figure 5A and 5B, exogenous Ang II triggered neuronal losses in the right SN of rats, as Ang II (60 ng/h) significantly decreased the number of TH-positive neurons by approximately 70% (*P* < 0.05). As seen in Figure 5A and 5B, co-treatment with losartan (200 ng/h) significantly rescued the decrease of TH-positive neurons caused by Ang II (*P* < 0.05). It should be noted that losartan (200 ng/h) alone did not significantly influence the number of TH-positive neurons in comparison to vehicle (*P* > 0.05). To further determine whether the loss of dopaminergic neurons was attributed to cell apoptosis, the activity of caspase-3, a key executor of apoptosis, was determined using a colorimetric assay kit. As shown by Figure 5C, Ang II (60 ng/h) increased the caspase-3 activity nearly by 2.5-fold (*P* < 0.05) of control, whereas co-infusion with losartan (200 ng/h) notably abolished this increase caused by Ang II. Taken together, these results demonstrated that exogenous Ang II triggers apoptosis of dopaminergic neurons *via* an AT1R-dependent manner in the right SN of rats.

### Oral administration of a novel AT1R blocker azilsartan ameliorates the apoptosis of dopaminergic neurons and rescues characteristic parkinsonian behaviors in a rat model of PD

To translate the above findings into a clinically relevant context, rats were orally administered with vehicle or a novel AT1R blocker azilsartan (5 mg/kg/d) during the four-week rotenone infusion (2.5 mg/kg/d). During this process, the SBP of rats was not significantly altered (Figure 2).

**Figure 6:**
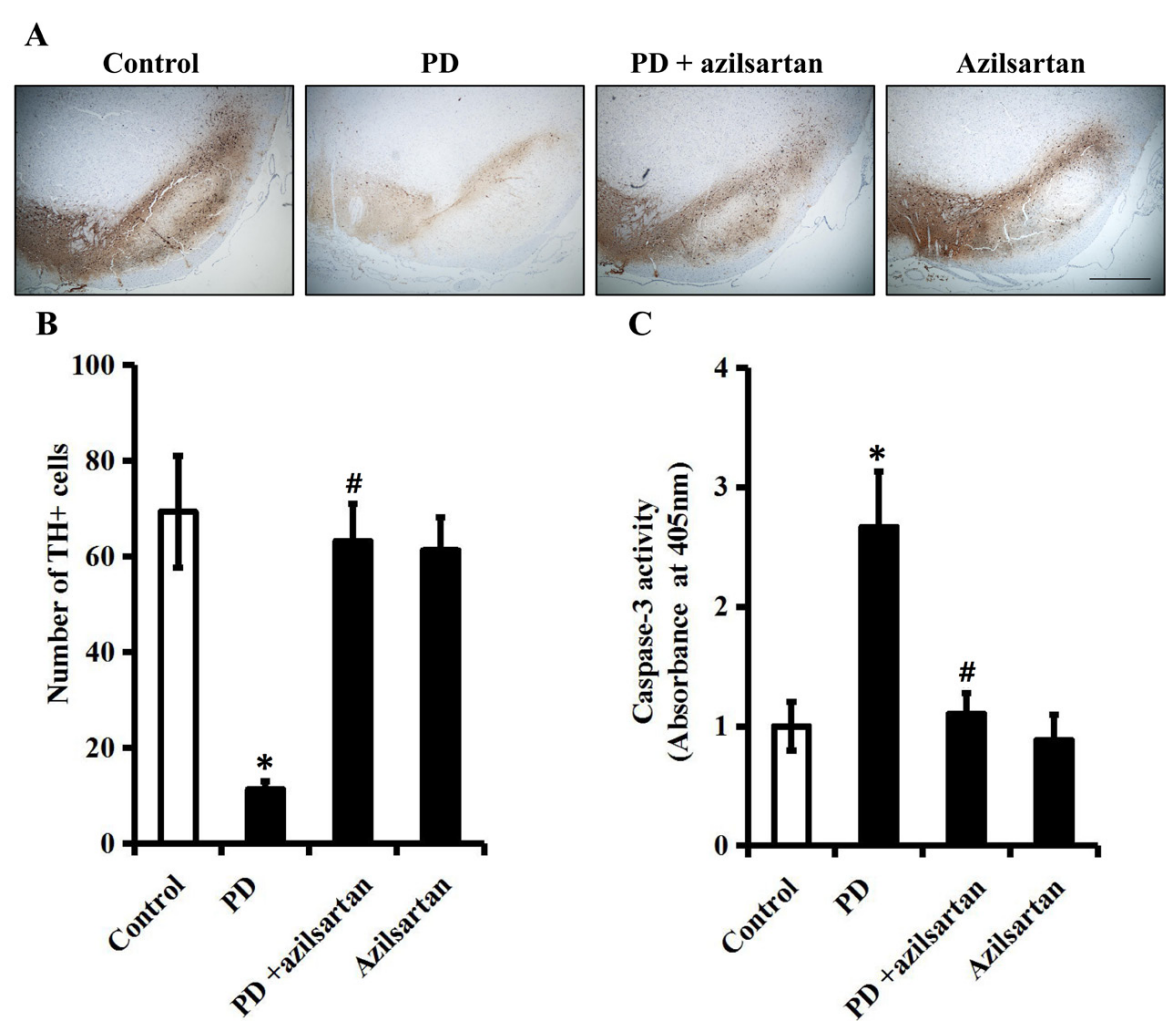
Oral administration of a novel AT1R blocker azilsartan ameliorates the apoptosis of dopaminergic neurons Rats were orally administered with vehicle or azilsartan (5 mg/kg/d) during the four-week rotenone (2.5 mg/kg/d) infusion. **A**. TH-immunoreactive neurons in the SN of each group. **B**. Quantitative analysis of TH-positive neurons in the SN of each group. Scale bar: 500 μm. **C**. The activity of caspase-3 in the SN was measured by a colorimetric assay kit. *n* = 6 per group. Data were analyzed by one-way ANOVA followed by Tukey's post hoc test. Columns represent mean±SD. *P < 0.05 *vs*. control group, #*P* < 0.05 *vs*. PD group.

As shown in Figure 6A and 6B, the decreased number of TH-positive neurons in the SN of PD rats was dramatically rescued by azilsartan (5 mg/kg/d) administration (*P* < 0.05). Of note, azilsartan (5 mg/kg/d) alone did not obviously change the number of TH-positive neurons in the SN of PD rats. The result indicated that azilsartan rescued the loss of dopaminergic neurons. As seen in Figure 6C, co-treatment with azilsartan (5 mg/kg/d) also attenuated the activation of caspase-3 in the SN of PD rats, since its activity was reduced by 1.6-fold (*P* < 0.05) after azilsartan treatment. This further indicated that azilsartan prevented neuronal losses *via* inhibiting apoptotic signaling.

**Figure 7:**
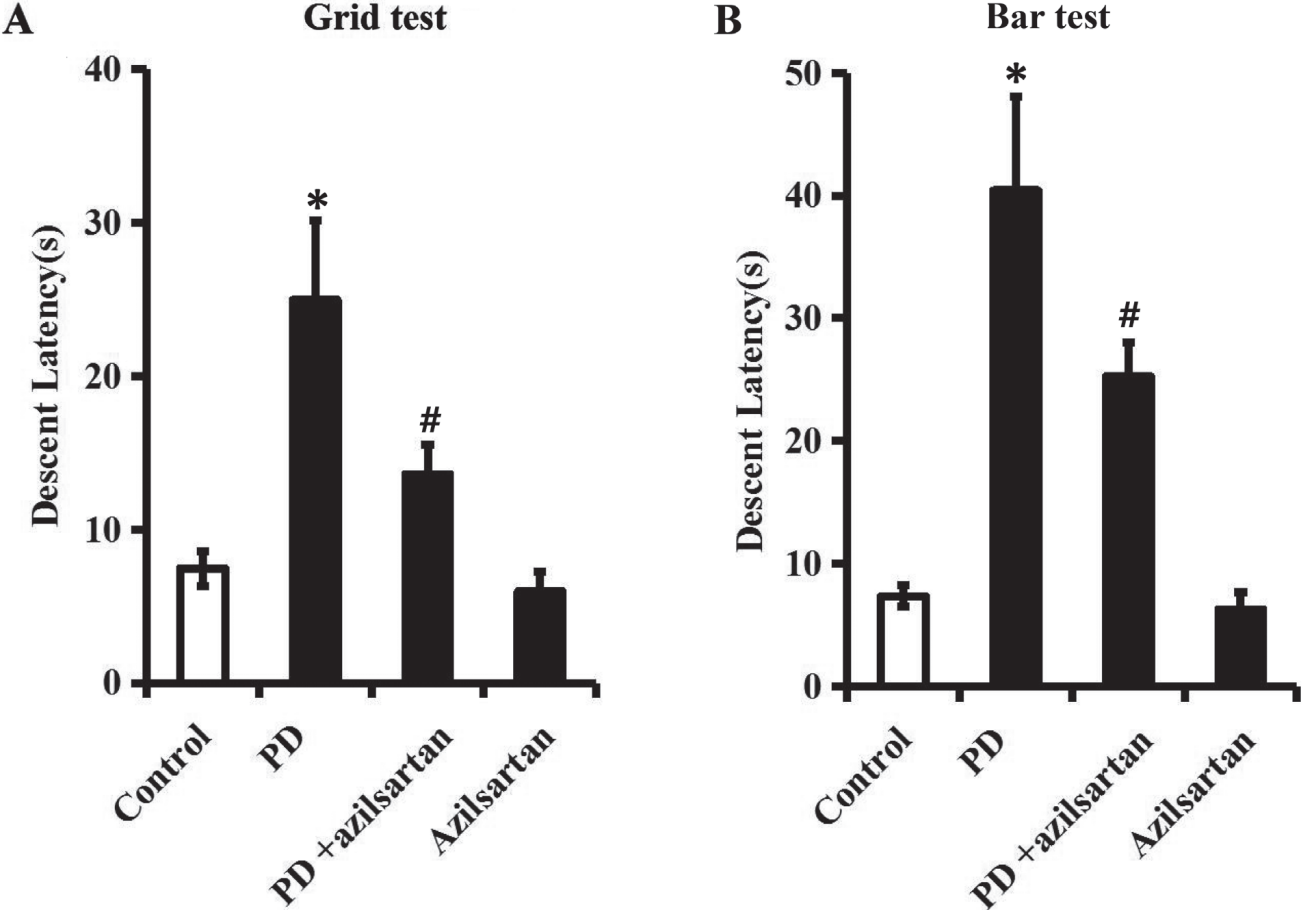
Azilsartan rescues characteristic parkinsonian behaviors in a rat model of PD Rats were orally administered with vehicle or azilsartan (5 mg/kg/d) during the four-week rotenone (2.5 mg/kg/d) infusion. **A**. and **B**. Descent latency of each group in the grid test and the bar test was recorded. *n* = 6 per group. Data were analyzed by one-way ANOVA followed by Tukey's post hoc test. Columns represent mean±SD. *P < 0.05 *vs*. control group, #*P* < 0.05 *vs*. PD group

Afterwards, the catalepsy tests were employed to investigate whether azilsartan can relieve the characteristic symptoms of PD rats. As suggested in Figure 7A and 7B, the prolonged descent latency of PD rats was significantly shortened following azilsartan (5 mg/kg/d) treatment (*P* < 0.05), indicating that azilsartan rescued characteristic parkinsonian behaviors in PD rats. It was noteworthy that azilsartan (5 mg/kg/d) had no obvious influence on the descent latency when compared with the control group (*P* > 0.05).

## DISCUSSION

Several lines of evidence suggested a potential association of Ang II/AT1R axis with the progression of PD, since a significantly higher level of Ang II in the CSF or brain tissues was observed in PD animal models as well as patients with this disease [13, 14]. Here, in support of these findings, we showed that the level of Ang II in the SN was obviously elevated in a rotenone-induced rat model of PD. Meanwhile, we also showed that AT1R level was increased in the SN of PD rats, and this was consistent with previous observations in an animal model of PD with dopamine depletion [15]. All these findings further strengthened the association between overactivation of Ang II/AT1R axis and development of PD.

Next, to further elucidate the role of brain Ang II/AT1R axis in PD progression, we directly infused exogenous Ang II into the SN of normal rats. We observed that excessive Ang II triggered an obvious loss of dopaminergic neurons in the SN of rats, since the number of TH-positive neurons was dramatically reduced. Meanwhile, we revealed the Ang II-induced loss of dopaminergic neurons was at least partially attributed to cell apoptosis, as the activity of caspase-3, a key executioner of apoptosis, was notably enhanced following Ang II infusion. Furthermore, the aforementioned pathogenic effects of Ang II in the SN of rat could be significantly reversed by co-infusion of losartan, an AT1R blocker. These findings supported our previous observations that Ang II triggered neuronal apoptosis *via* an AT1R-dependent manner in dopaminergic neuronal cell lines or mouse primary dopaminergic neurons [9, 16, 17]. Intriguingly, the underlying mechanisms by which Ang II triggered apoptosis of dopaminergic neurons seemed to be multifactorial. Previously, using CATH. a cell, a dopaminergic neuronal cell line stably expressing AT1R, we demonstrated that Ang II triggered apoptosis *via* enhancement of NADPH oxidase-mediated oxidative stress [9]. Meanwhile, another *in vitro* study indicated that Ang II induced apoptosis of dopaminergic neurons by activation of autophagy through an AT1R-mediated fashion [16]. In addition, emerging evidence suggested that mitochondrial-dependent apoptotic signaling was also involved in Ang II-induced apoptosis in dopaminergic neurons [17]. Nevertheless, these potential mechanisms needed to be further verified *in vivo* using PD animal models.

It is worthy to note that our findings also implied a therapeutic potential of AT1R blocker for PD treatment. As a newly developed AT1R blocker, azilsartan was approved by FDA for the treatment of essential hypertension in 2011 [18]. According to a receptor binding and function study, azilsartan bound tightly to and dissociated slowly from AT1R when compared with other antagonists, and thus elicits more potent and long-lasting bioeffects *in vivo* [19]. Apart from its main applications, azilsartan was revealed to exert beneficial effects in animal models of myocardial infarction [20]. Meanwhile, azilsartan was able to ameliorate metabolic syndrome and kidney damage in rats with type 2 diabetes [21]. In addition, anti-inflammatory actions of oral azilsartan treatment was also observed in an experimental model of oral mucositis [22]. Interestingly, in this study, we extended its application by showing that oral administration of azilsartan for 4 weeks not only rescued the apoptosis of dopaminergic neurons but also relieved the characteristic parkinsonian symptoms in a PD rat model induced by rotenone. Of note, these effects are independent of its BP-lowering effects, since azilsartan at the current dose (5mg/kg/d) and treatment duration (4 weeks) did not significantly alter the SBP of PD rats. To our knowledge, this is the first study demonstrating a protective effect of chronic azilsartan administration in a rat model of PD.

Summarily, in the current study, we provide *in vivo* evidence that overactivation of brain Ang II/AT1R axis contributes to the progression of PD. More importantly, we reveal a protective effect of azilsartan, a newly developed AT1R blocker, in a rat model of PD induced by rotenone. These results further support the application of AT1R blockers in the therapy of PD, and strengthen the notion that many therapeutic agents may possess pleiotropic action in addition to their main applications [23, 24].

## MATERIALS AND METHODS

### Reagents and preparation

Ang II, losartan (an AT1R antagonist), and rotenone were purchased from Sigma-Aldrich Inc. Azilsartan (a novel AT1R antagonist) was purchased from MedChem Express Inc.

### Animals and experimental groups

Male Lewis rats (280-300 g) were obtained from the Shanghai branch of the National Rodent Laboratory Animal Resources of China. Rats were housed in an air-conditioned room under 12 h light and dark cycles. Water and food were provided *ad libitum*. Animal care and experimental protocols were carried out in accordance with the Guide for the Care and Use of Laboratory Animals of Nanjing First Hospital and were approved by the Biological Research Ethics Committee of Nanjing First Hospital.

A rat model of PD was established as described [12]. Osmotic minipumps (model 2ML4, Alzet Inc.) were filled with rotenone or vehicle alone. Pumps were incubated at 37°C overnight in sterile saline before implantation. Rats were anesthetized with 10% chloral hydrate (0.35 mL/100 g), and osmotic minipumps were embedded under the skin of the back for 4 weeks. Control rats received vehicle, and treated rats received rotenone (2.5 mg/kg/day).

To investigate the relationship between Ang II/AT1R axis and PD pathogenesis, male Lewis rats were randomly divided into 4 groups: control group, Ang II (60 ng/) group, Ang II (60 ng/h) + losartan (200 ng/h) group, and losartan (200 ng/h) group. The doses of Ang II and losartan were chosen according to our previous studies [25, 26]. They were continuously infused into the right SN, alone or in combination, for one week by a osmotic pump with an indwelling cannula (model 2004, Alzet Inc.) [25, 27]. The details regarding supranigral infusion were described in section 4.3.

To determine whether oral administration of azilsartan exerts neuroprotection in PD models, rats were randomly divided into 4 groups: control group, PD group, PD + azilsartan (5 mg/kg/d, i.g., for 4 weeks) group and control + azilsartan (5 mg/kg/d, i.g., for 4 weeks) group. The dose of azilsartan was selected based on a recent study by Hye et al [28].

### Supranigral infusion

Briefly, a 7-day unilateral right supranigral infusion was performed with osmotic pumps (model 2004, Alzet Inc.) connecting with an indwelling cannula placed above the dorsal mid-portion of the right SN as described [27]. Before implanted into animals, the pumps were incubated in sterile saline for at least 40 h at 37°C. Rats were fixed to a stereotactic frame (David Kopf Inc.) after being anesthetized with 10% chloral hydrate (0.35 ml/100 g). Stainless infusion cannula was implanted in predetermined coordinates for the right supranigral placement (anteroposterior: 5.5 mm; mediolateral: 2.5 mm; dorsoventral: 7.0 mm, from bregma as a reference). The pumps were embedded subcutaneously on the backs of the rats.

### Blood pressure measurements

SBP was measured by the tail cuff method (Visitech Systems Inc.) at the end of treatments as described [29]. Each measurement was carried out 3-5 times to acquire a mean SBP.

### Neurological behavioral measurements (catalepsy tests)

The catalepsy tests contained a vertical grid test and a horizontal bar test. They were performed at the end of treatments as described [30].

For the vertical grid test, a specific gridiron (44 cm high and 25.5 cm wide with a space of 1 cm between wires) was used. When the rat was hung by all four paws on the vertical grid, the stopwatch was started. When a paw dropped from the grid, the stopwatch was stopped. The taken time was recorded as descent latency. Maximum descent latency time was fixed at 120 s.

For the horizontal bar test, each rat was placed with its forepaws on a metal rod suspended 9 cm above the floor. The taken time (descent latency) was recorded before it climbed down from the bar. Maximum descent latency time was fixed at 120 s.

### EIA for Ang II levels in the SN

The SN of rat was dissected and homogenized, then centrifuged (3000 r/min for 15 min at 4°C) to remove cellular debris. The level of Ang II in the SN was measured using specific EIA kits (Phoenix Pharmaceuticals Inc.) as described [29]. The absorbance of every well was determined by the spectrophotometer at 450 nm.

### Colorimetric assay for caspase-3 activity

The activity of caspase-3 in the SN of rat was measured by a colorimetric assay as described [17]. In brief, the SN of rats were lysed in extraction buffer (Beyotime Inc.), and the activity of caspase-3 was analyzed with a colorimetric assay kit (Abcam Inc.) following the manufacturer's instructions.

### qRT-PCR

Total RNA was extracted from the SN with Trizol (Invitrogen, Inc.) according to the manufacturer's guidelines. Equal amounts of total RNA were reverse transcribed with the PrimeScript^TM^ RT Master Mix (Takara Bio Inc) under standard conditions as described [31]. Subsequently, quantitative real-time PCR reactions were performed with SYBR Green Premix Ex Taq (Takara Bio Inc) and specific primers to detect AT1R and AT2R expressions. Meanwhile, glyceraldehyde 3-phosphate dehydrogenase (GAPDH) was adopted as an internal control, as its expression showed minimal variation in different tissues. Primers sequences: for AT1R: forward 5′-TTCAACCTCTACGCCAGTGTG-3′, reverse 5′-GCCAAGCCAGCCATCAGC-3′, for AT2R: forward 5′-AACATCTGCTGAAGACCAATAG-3′, reverse 5′-AGAAGGTCAGAACATGGAAGG-3′, for GAPDH: forward 5′-TGCCACTCAGAAGACTGTGG-3′, reverse 5′-TTCAGCTCTGGGATGACCTT-3′.

### Western blotting analysis

Western blotting analysis was performed as described [32]. The SN tissues were homogenized, the total proteins were extracted in RIPA lysis buffer (Beyotime Inc.) and the protein concentrations were assayed by a BCA kit (Beyotime Inc.). Different samples with an equal amount of protein were separated on 10-15% SDS polyacrylamide gels, transferred to nitrocellulose filter membranes, and blocked in 5% nonfat milk at room temperature for 2 h. The membranes were incubated overnight at 4°C with primary antibodies against AT1R (1:800; Abcam Inc.), AT2R (1:1000; Abcam Inc.) and β-actin (1:500; Santa Cruz Biotechnology Inc.). Afterwards, the membranes were washed with 1×TBST for three times and incubated with horseradish peroxidase (HRP)-coupled secondary antibody for 2 h at room temperature. Finally, after rinsing with 1×TBST, protein bands were detected with the chemiluminescent HRP substrate (Thermo Scientific Inc.) for 5min at room temperature and exposed to X-ray film (Fujifilm Inc.). The signal intensity was quantified using Quantity One software 4.6.2 (Bio-Rad Laboratories Inc.) and normalized to β-actin.

### Immunohistochemical staining

Rats were sacrificed under deep anesthesia and the brains were removed and placed in 4% paraformaldehyde solution overnight. Coronal sections (from 24.5mm to 26.2mm, caudal to the bregma, which contains the SN) were prepared by a sledge microtome [12]. The SN sections were dewaxed, hydrated and treated with 0.3% H_2_O_2_ for 30 min to quench the endogenous peroxidases. Then the slides were dealt with 0.5% Triton-X 100 for 30 min, blocked with 5% bovine serum albumin for 30 min, and incubated at 4°C overnight with primary antibodies: monoclonal mouse anti-tyrosine hydroxylase (anti-TH; 1:2000; Sigma Inc.). After that the sections were incubated for 1 h in the secondary antibody: peroxidase conjugated goat anti-mouse IgG (1:1000; Sigma Inc.). After the diaminobenzidine reaction, sections were counterstained with Mayer's hematoxylin, dehydrated, and mounted on slides. Cells with positive tyrosine hydroxylase immunoreactivity (brown granules) were counted as TH-positive cells. Cell counting was performed with a microscope equipped with a camera by three independent observers who were blinded to the experimental groups.

### Statistical analysis

Data were presented as mean±SD. Comparisons between groups were assessed by independent samples t test or one way analysis of variance (ANOVA) followed by Tukey's post hoc test using SPSS software (version 17.0). Differences were considered significant at *P* < 0.05.
